# A novel and convenient method to evaluate bone cement distribution following percutaneous vertebral augmentation

**DOI:** 10.1038/s41598-020-73513-2

**Published:** 2020-10-01

**Authors:** Jin Liu, Jing Tang, Hao Liu, Zuchao Gu, Yu Zhang, Shenghui Yu

**Affiliations:** 1 Department of Orthopaedics, Sichuan University West China Hospital, Chengdu, China; 2 Department of Orthopaedics, Chengdu First People’s Hospital, Chengdu, China; 3 Department of Radiology, Sichuan University West China Hospital, Chengdu, China

**Keywords:** Diagnosis, Disease prevention, Fracture repair, Medical imaging, Prognosis, Therapeutics, Outcomes research

## Abstract

A convenient method to evaluate bone cement distribution following vertebral augmentation is lacking, and therefore so is our understanding of the optimal distribution. To address these questions, we conducted a retrospective study using data from patients with a single-segment vertebral fracture who were treated with vertebral augmentation at our two hospitals. Five evaluation methods based on X-ray film were compared to determine the best evaluation method and the optimal cement distribution. Of the 263 patients included, 49 (18.63%) experienced re-collapse of treated vertebrae and 119 (45.25%) experienced new fractures during follow-up. A 12-score evaluation method (kappa value = 0.652) showed the largest area under the receiver operating characteristic curve for predicting new fractures (0.591) or re-collapse (0.933). In linear regression with the 12-score method, the bone cement distribution showed a negative correlation with the re-collapse of treated vertebra, but it showed a weak correlation with new fracture. The two prediction curves intersected at a score of 10. We conclude that an X-ray-based method for evaluation of bone cement distribution can be convenient and practical, and it can reliably predict risk of new fracture and re-collapse. The 12-score method showed the strongest predictive power, with a score of 10 suggesting optimal bone cement distribution.

## Introduction

Since Galibert and Deramond first used bone cement to treat aggressive cervical hemangiomas in 1987^1^, this procedure has been verified by numerous studies to be an effective minimally invasive surgery for the treatment of osteoporotic vertebral compression fractures (OVCFs)^2–5^. As the population continues to age, the incidence of vertebral compression fractures caused by osteoporosis has also increased^6–8^. For the foreseeable future, vertebral augmentation will continue to play an important role as a minimally invasive treatment of OVCFs.

Vertebral augmentation can quickly alleviate the pain caused by OVCFs only when the bone cement rapidly halts crack propagation of the fracture and restores the height and stiffness of the fractured vertebra to meet weight-bearing demand. In addition, cytotoxicity and heat generation of bone cement also play a role in pain relief of patients received vertebral augmentation^9–11^. The amount of bone cement used can influence risk of various complications after vertebral augmentation^12–21^. Injection of excessive bone cement tends to induce new fractures of adjacent vertebral bodies and may increase the leakage rate^12–15^, while too little bone cement can result in insufficient filling, which can fail to relieve the pain rapidly^15,16^ and easily induce re-collapse of the treated vertebrae^17–19^. However, the amount of bone cement per se is unlikely to be the most reliable predictor of whether the cement will achieve a satisfactory distribution, given differences in vertebral body size as well as in location and degree of compression^14–16^.

Many studies have shown the distribution of bone cement to be a more suitable index^12,13,18,19,22–24^. Calculating the volume fraction of bone cement based on computed tomography may be more accurate, while computer simulation combined with hydrodynamics can also model distribution of bone cement^25–27^. However, these methods are quite complex and cannot be conducted in real time to guide intraoperative procedures^14–16^. Methods based on X-ray images have been reported to evaluate distribution of bone cement, but they differ widely and lack consistency or prognostic relevance^12,15,22–24^. Consequently, there is no recognized, practical way to evaluate the distribution of bone cement in vertebral augmentation and therefore predict risk of subsequent re-collapse or new fracture.

To address this gap, we assessed different methods for analyzing the distribution of bone cement on X-ray images after vertebral augmentation, and then predicting new symptomatic fracture or re-collapse of augmented vertebrae. Our goal was to determine the best evaluation method and the corresponding optimal distribution of bone cement.

## Materials and methods

### Patient characteristics

This study was approved by the Ethics Committee of West China Hospital of Sichuan University. All methods and procedures were carried out in accordance with relevant guidelines and regulations, and informed consent was obtained from all subjects. This retrospective study included data from patients who received vertebral augmentation in our two institutions from April 2014 to March 2019. The specific inclusion criteria were: (1) the patient was aged ≥ 70 years, or the bone mineral density (BMD) measured by dual-energy X-ray absorptiometry was T ≤ -2.5; (2) the patient had a known history of hypochondriac pain and/or back pain, with or without limited mobility; (3) acute, single-segment OVCF was confirmed by magnetic resonance imaging (MRI); and (4) the patient was followed up for at least 6 months after surgery, with the most recent X-ray examination at least 6 months postoperatively. Patients were excluded if they underwent vertebral augmentation because of pathological fractures caused by spinal neoplasms, or if they suffered new fractures caused by high-energy trauma during the follow-up period.

### Surgical procedure

All patients were given local anesthesia, and surgery was performed under a C-arm X-ray machine while the patient was lying in the prone position. A 3.5-mm puncture needle was used to puncture the pedicle of the vertebra. During the needle insertion, the angles of introversion, extroversion, and cephalocaudal inclination were adjusted based on C-arm fluoroscopy, until the puncture needle reached the junction of the middle and posterior thirds of the vertebral body. Then, if the patient chose percutaneous kyphoplasty (PKP), the balloon was inserted into the vertebra with height reduction, followed by removal of the balloon and rapid injection of polymethylmethacrylate. If the patient chose percutaneous vertebroplasty (PVP), direct polymethylmethacrylate injection was performed. The same kind of bone cement (Osteopal V, Germany) was used in all patients regardless of procedure method.

### Post-operative treatment and follow-up

After surgery, patients rested in the supine position for 3 h and gradually resumed off-bed activities with waist support. Routine examinations of anteroposterior and lateral X-ray films were performed to assess the distribution and leakage of bone cement. Computed tomography and MRI were performed on patients whose pain was exacerbated or not significantly alleviated, and on patients who had emerging nerve root pain; based on the findings, further treatment was undertaken as necessary. Patients were routinely administered calcium and active vitamin D after surgery, and 31 patients elected additional treatment with zoledronic acid.

After discharge, patients were followed-up by telephone every 3 months to enquire about their pain and daily activities. If a patient mentioned back or hypochondriac pain that had lasted longer than 3 days or was not relieved significantly after taking nonsteroidal anti-inflammatory drugs (NSAIDs), they were instructed to return to hospital for an X-ray examination. If new fractures were strongly suspected, confirmatory MRI was performed.

### Description of variables

Data were collected on each patient’s sex, age, body mass index (BMI), and puncture method (unilateral or bilateral), treatment method (PVP or PKP), cause of fracture, and other imaging data, including X-ray films before surgery and during follow-up. A spine surgeon (11 years of experience in spine surgery) and a radiologist (7 years of experience in musculoskeletal system images) jointly evaluated the images to determine the number of augmented vertebral bodies, whether there was a cleft in the fractured vertebra, location of the fractured vertebra, whether the degree of compression of the fracture exceeded 50%, re-collapse of the augmented vertebra, new fracture, and bone cement leakage. Five different evaluation methods were used to assess the bone cement distribution. When the evaluation results differed, the two physicians discussed and made a final decision.

**Figure 1:**
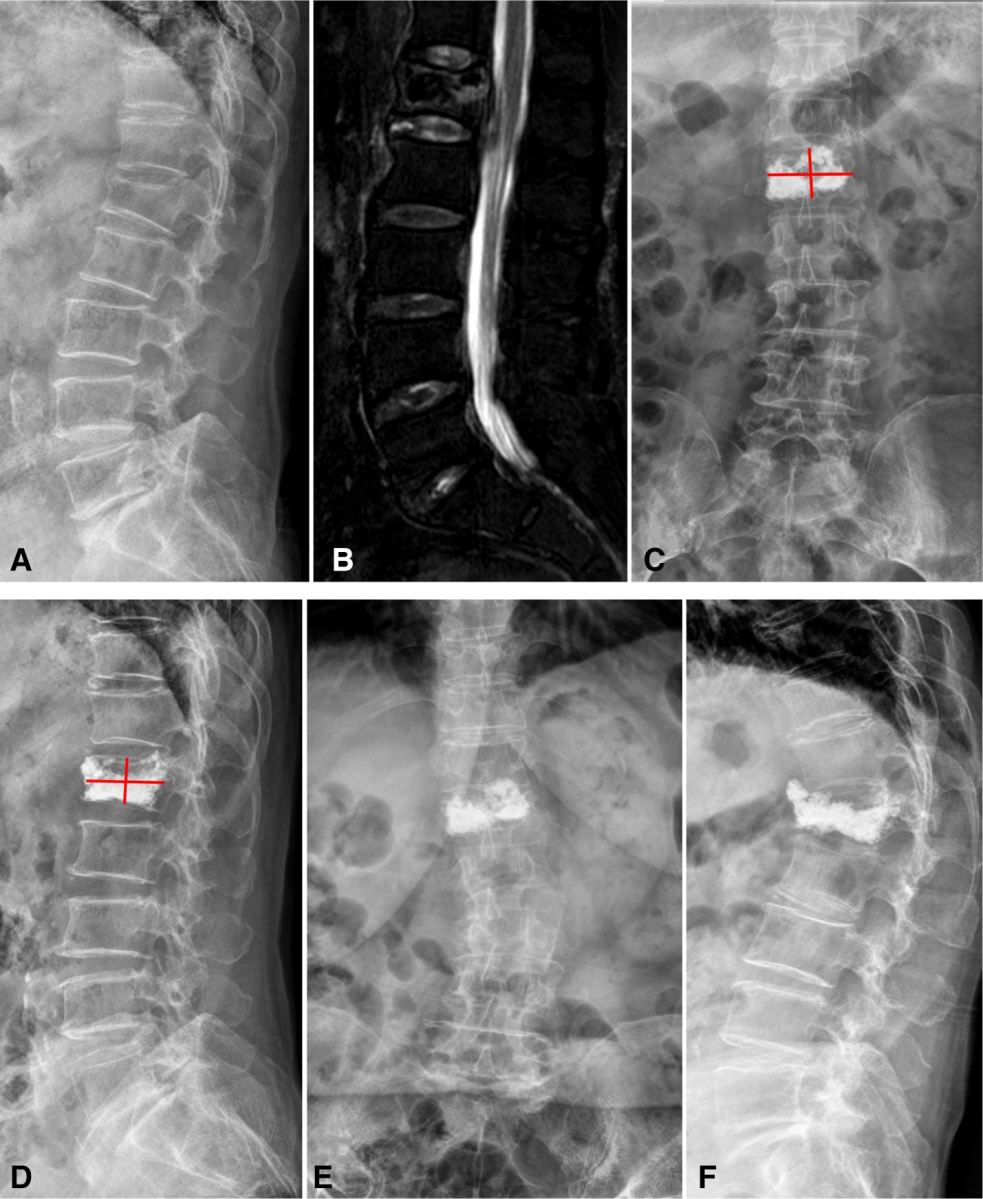
X-ray images of a 79-year-old woman. (**A**,**B**) Images taken at admission, showing vertebral compression fracture at L1. (**C**,**D**) Anteroposterior and lateral images taken after percutaneous kyphoplasty, showing restoration of the fracture. Bone cement distribution was scored as 7 using method 1, 8 using method 2, 6 using method 3, and 9 using methods 4 and 5. (**E**,**F**) Images taken at 6 months after surgery, showing re-collapse of the operated vertebra.

Re-collapse of augmented vertebrae was diagnosed when the angle between the upper and lower endplates of the augmented vertebra (based on images from the last follow-up) had changed by more than 10° from the angle immediately after surgery, or when the height of the augmented vertebra was compressed by more than 15% from the height immediately after surgery^19^. New fracture was diagnosed based on hyperintensity on T2-weighted MRI with fat suppression. A typical series of images is shown in Fig. 1.

Based on previous reports^15,23^, we developed and compared three new methods to evaluate anteroposterior and lateral X-ray images. Our goal was to identify a method showing good inter-rater consistency that could reliably predict new fracture and re-collapse. The methods differed in how vertebral quadrants were defined and in how affected quadrants were evaluated. We named the five methods based on the number of scores that each generated: method 1 was termed the 8-score method; method 2, 10-score; method 3, new 8-score; method 4, 9-score; and method 5, 12-score. Further details of the five methods are described in Table 1.

**Table 1:** Methods to evaluate cement distribution from X-ray images in this study.

Method	Source	Description		Scores
Method 1 (8-score)	Liu et al.^23^	Vertebra are divided into quadrants based on the anteroposterior and lateral positions. Quadrants are counted if the bone cement filling exceeds one-third of the quadrant		N = 4 + 4
Method 2 (10-score)	Newly designed	Based on the 8-score method described by Liu et al.^23^. Quadrants are counted if the bone cement contacts the upper or lower endplate as viewed in the lateral position. Each sign is considered to be independent effective quadrant		N = 4 + 4 + 2
Method 3 (new 8-score)	Newly designed	Vertebra are divided into quadrants based on the anteroposterior and lateral position. Quadrants are counted if the bone cement filling exceeds half of the quadrant		N = 4 + 4
Method 4 (9-score)	Sun et al.^15^	On the anteroposterior plain, a score of 3 (> 75%), 2 (50%-75%), 1 (25%-50%), or 0 (< 25%) is assigned based on the percentage of bone cement distribution across the width of the vertebra On the lateral plane, a score of 3 (> 75%), 2 (50%-75%), 1 (25%-50%), or 0 (< 25%) is assigned based on the percentage of bone cement distributed across the sagittal width and the vertical height of the vertebra respectively		N = 3 + 3 + 3
Method 5 (12-score)	Newly designed	Based on the new 8-score method. If the bone cement contacts the upper or lower endplate of the vertebra in the lateral plane, or if the bone cement crosses the midline on the anteroposterior or lateral plane, then each sign is considered to be an independently effective quadrant		N = 4 + 4 + 2 + 2

### Statistical analyses

Measurements were expressed as mean ± standard deviation. Inter-group differences in continuous variables were assessed for significance using one-way ANOVA in the case of normally distributed data, or the Kruskal–Wallis rank-sum test in the case of skewed data. Differences in categorical variables were assessed using the χ^2^ test. The kappa statistic was used to assess agreement between the two clinicians in assessing bone cement distribution, while receiver operating characteristic (ROC) curves were calculated to assess ability to predict new fracture or re-collapse of augmented vertebrae. The bone cement evaluation method that had the largest area under the ROC curve (AUC) was used to plot the cumulative curves of new fracture and re-collapse at each score, and linear regression was performed in each case. Statistical analyses were performed using SPSS 23.0 (IBM, Armonk, New York, USA) at a significance level of α = 0.05.

## Results

### Patient characteristics and rates of new fracture and re-collapse

A total of 263 patients with single-segment OVCFs were enrolled in this study, including 43 males and 220 females, with an average age of 73.43 ± 8.34 years. Among them, 62 patients had a history of vertebral augmentation. A total of 137 new fractures were observed in 119 (45.25%) of the 263 patients, of which 54 (45.38%) were adjacent or included adjacent vertebrae. There were significant differences between patients with or without new fractures in terms of distribution of bone cement, thoracolumbar location, age, number of augmented vertebrae, and whether clefts were present or the cause of the fracture was known (Table 2). During follow-up, 49 of 263 (18.63%) patients suffered re-collapse of augmented vertebrae. There were significant differences between patients with or without vertebral re-collapse in terms of puncture method, thoracolumbar location, number of augmented vertebrae, and bone cement distribution (Table 2). One patient died during the follow-up period.

**Table 2:** Baseline characteristics of patients, stratified by whether they experienced new fractures or re-collapse of the augmented vertebra.

	New fracture				Re-collapse			
	Yes (n = 119)	No (n = 144)	Statistical value	P	Yes (n = 49)	No (n = 214)	Statistical value	P
Age (years)	75.65 ± 0.63	71.60 ± 0.75	W = 16,756.500^△^	< 0.001*	74.63 ± 8.87	73.15 ± 8.21	F = 1.254	0.264
Number of augmented vertebrae	0.79 ± 0.10	0.11 ± 0.03	W = 16,024.000^△^	< 0.001*	0.18 ± 0.09	0.47 ± 0.06	W = 5614.000^△^	0.017*
BMI (kg/m^2^)	22.29 ± 3.40	23.21 ± 4.06	F = 3.384	0.051	22.56 ± 2.74	22.85 ± 4.00	F = 0.229	0.633
**Sex**								
Male	15	28	χ^2^ = 2.228	0.206	11	32	χ^2^ = 1.638	0.201
Female	104	116			38	182		
**Zoledronate**								
Yes	15	16	χ^2^ = 0.140	0.708	6	25	χ^2^ = 0.012	0.912
No	104	128			43	189		
**Fracture cause known**								
Yes	40	88	χ^2^ = 19.720	< 0.001*	29	99	χ^2^ = 2.665	0.103
No	79	56			20	115		
**PVP or PKP**								
PVP	80	89	χ^2^ = 0.834	0.361	27	142	χ^2^ = 2.198	0.138
PKP	39	55			22	72		
**Puncture method**								
Unipedicular	17	27	χ^2^ = 0.932	0.334	17	27	χ^2^ = 13.949	< 0.001*
Bipedicular	102	117			32	187		
**Cleft sign**								
Yes	22	47	χ^2^ = 6.742	0.009*	18	51	χ^2^ = 3.430	0.064
No	97	97			31	163		
**Severity of compression**								
< 50%	102	123	χ^2^ = 0.005	0.946	40	185	χ^2^ = 0.748	0.387
≥ 50%	17	21			9	29		
**Thoracolumbar segments (T11–L3)**								
Yes	72	113	χ^2^ = 10.083	0.001*	44	141	χ^2^ = 10.924	0.001*
No	47	31			5	73		
**Bone cement leakage**								
Yes	53	71	χ^2^ = 0.594	0.441	22	102	χ^2^ = 0.122	0.726
No	66	73			27	112		
**Bone cement distribution**								
Method 1	7.25 ± 0.98	6.97 ± 1.13	F = 4.495	0.035*	5.96 ± 0.17	7.36 ± 0.06	W = 3130.000^△^	< 0.001*
Method 2	9.09 ± 1.17	8.72 ± 1.36	F = 5.460	0.020*	7.29 ± 0.19	9.26 ± 0.07	W = 2544.000^△^	< 0.001*
Method 3	6.88 ± 1.12	6.56 ± 1.21	F = 5.074	0.025*	5.22 ± 0.96	7.04 ± 0.94	F = 147.447	< 0.001*
Method 4	8.56 ± 0.07	8.25 ± 0.08	W = 17,606.000^△^	0.009*	7.45 ± 0.15	8.61 ± 0.05	W = 3247.000^△^	< 0.001*
Method 5	10.60 ± 1.55	10.11 ± 1.66	F = 5.940	0.015*	8.02 ± 1.27	10.86 ± 1.17	F = 227.504	< 0.001*

*Statistically significant.

^△^Indicates that data were skewed, so the Kruskal–Wallis test was used.

### Inter-rater consistency and ROC curves for different evaluation methods

**Figure 2:**
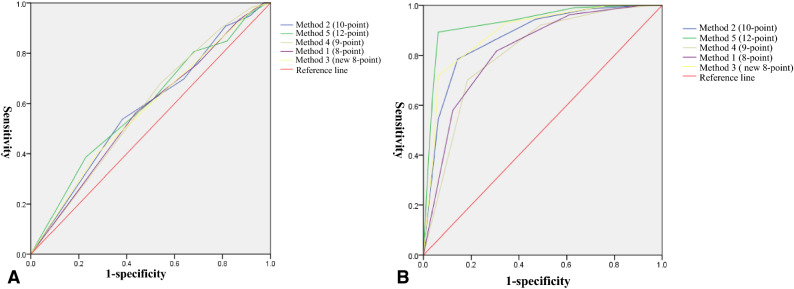
Receiver operating characteristic curves to assess the ability of the various methods of bone cement evaluation to predict (**A**) new fracture or (**B**) re-collapse of the operated vertebra.

Two physicians used five methods to assess bone cement distribution based on X-ray images. The kappa values were as follows: 8-score, 0.716; 10-score, 0.695; new 8-score, 0.673; 9-score, 0.714; and 12-score, 0.652 (Table 3). The corresponding AUCs for predicting new fractures were 0.568, 0.579, 0.576, 0.582, and 0.591 (Fig. 2A). Except for the 8-score method (P = 0.059), all other methods showed statistical significance (P < 0.05, Table 3). Respective AUCs for predicting incidence of augmented vertebra collapse were 0.818, 0.874, 0.893, 0.807, and 0.933 (Fig. 2B), which were all statistically significant (P < 0.05, Table 3). The 12-score method showed higher AUCs for predicting new fractures and re-collapse of augmented vertebrae (Table 3).

**Table 3:** Inter-rater consistency and ability of each bone cement evaluation method to predict new fracture or re-collapse of augmented vertebra.

Method	Consistency		Re-collapse		New fracture	
	Kappa value	P value	AUC	P value	AUC	P value
Method 1 (8-score)	0.716	0.033	0.818	< 0.001	0.568	0.059^#^
Method 2 (10-score)	0.695	0.033	0.874	< 0.001	0.579	0.027
Method 3 (New 8-score)	0.673	0.034	0.893	< 0.001	0.576	0.035
Method 4 (9-score)	0.714	0.037	0.807	< 0.001	0.582	0.022
Method 5 (12-score)	0.652	0.033	0.933	< 0.001	0.591	0.011

*AUC* area under the receiver operating characteristic curve.

^#^Not statistically significant.

### Predictive value of 12-score method based on linear regression

**Figure 3:**
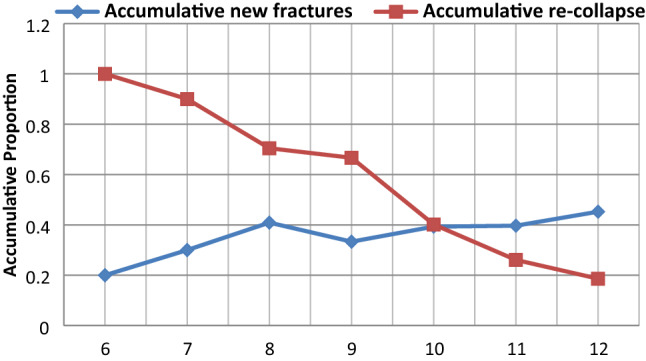
Cumulative proportions of new fracture and re-collapse of treated vertebra associated with each score observed in our patient sample based on the 12-score method.

Scores for the 12-score method concentrated in the range of 6–12, with a mean of 10.33 ± 1.62. The cumulative proportions of new fractures and re-collapse corresponding to each score are shown in Table 4, and the line chart based on these data is shown in Fig. 3. The score corresponding to the intersection point of the two lines was 10. Linear regression showed that the distribution of bone cement was a significant predictor of re-collapse of the operated vertebra (F = 237.753, P < 0.001; Table 5), with a regression coefficient of − 0.144 [95% confidence interval (CI) − 0.168 to − 0.120], and a significant predictor of new fracture (F = 13.091, P = 0.015; Table 5), with a regression coefficient of 0.033 (95% CI 0.010–0.057).

**Table 4:** Cumulative numbers of new fractures and re-collapses of augmented vertebrae for each score observed using the 12-score method.

Score	Cumulative number of cases of new fracture	Cumulative number of total cases	Cumulative propotion of new fractures	Cumulative number of cases of re-collapse	Cumulative number of total cases	Cumulative propotion of re-collapse
6	1	5	0.2000	5	5	1.0000
7	6	20	0.3000	18	20	0.9000
8	18	44	0.4091	31	44	0.7045
9	23	69	0.3333	46	69	0.6667
10	46	117	0.3932	47	117	0.4017
11	73	184	0.3967	48	184	0.2609
12	119	263	0.4525	49	263	0.1863

**Table 5:** Linear regression analysis of the 12-score method’s predictions of new fracture or re-collapse of the treated vertebra.

Prediction	Parameter	Regression coefficient	95% confidence interval		F value	P
			Lower limit	Upper limit		
New fracture	Constant	0.0540	− 0.164	0.273	13.091	0.015*
	Bone cement distribution	0.0330	0.01	0.057		
Re-collapse	Constant	1.1881	1.661	2.102	237.753	< 0.001*
	Bone cement distribution	− 0.1440	− 0.168	− 0.12		

*Statistically significant.

## Discussion

The distribution of bone cement is known to affect the outcome and complications of vertebral augmentation^12–22^, but there is no recognized, convenient, effective method to assess such distribution or predict prognosis for patients undergoing vertebral augmentation. It would be particularly helpful if the method could assess distribution and predict outcome in real time to guide surgery. In the present study, we compared the ability of five methods to assess bone cement distribution from X-ray images and predict risk of new fracture or re-collapse of treated vertebrae. The methods here were taken directly from the literature (8-score method^23^ and 9-score method^15^) or newly developed (new 8-score method, 9-score method and 12-score method). Among the five methods, the newly designed 12-score method showed high inter-rater consistency and strongest ability to predict new fracture and re-collapse of augmented vertebrae. Our results suggest that the distribution of bone cement correlates negatively with re-collapse of augmented vertebrae and positively but weakly with new fracture. In this scoring system, a score of 10 appears to indicate the best balance between preventing new fracture and re-collapse of augmented vertebrae.

Bone cement injected during vertebral augmentation is commonly measured in terms of amount and morphological distribution^12–19,22^. However, the amount of bone cement stored in the fractured vertebrae alone is not an ideal index because of variations in vertebral body size and OVCF severity^14–16^. Although computed tomography is theoretically the most accurate method to calculate the ratio of bone cement volume to vertebral volume^14–16^, it is complex and inconvenient for intraoperative use, and it does not take into account morphological features^13,19,24^. In contrast, methods based on X-ray images can show whether the bone cement touches the upper and lower endplates^22^, whether it crosses the midline and is continuous^12,24^, and the amount of bone cement deposited^12,13,15,16^; all these factors are related to clinical outcomes. So far, however, methods based on X-ray images have not been assessed in terms of inter-rater consistency, sensitivity, or specificity.

All five evaluation methods for bone cement distribution based on X-ray imaging showed good consistency in this study. Although the inter-rater consistency of the 12-score method in this study was not as good as that of other methods, as indicated by kappa values 0.652, it had the largest AUC in predicting re-collapse of treated vertebrae and new fractures. This method takes into account the morphological characteristics of bone cement in all directions as well as the degree of filling, which may help explain its high prediction ability. Linear regression based on the 12-point method showed that bone cement distribution had a nearly negative correlation with re-collapse of augmented vertebra, but a weak positive correlation with new fracture. The two curves intersected at a score of 10, indicating that patients with this score are at lowest risk of re-collapse and new fracture. This score may be a clinically useful target to achieve, as a complement or alternative to the target of 19.78–25% bone cement volume fraction suggested by some studies^14–16^. The score could be determined intraoperatively using C-arm radiography.

Our study further shows that the distribution of bone cement affects risk of re-collapse of augmented vertebra and new fracture. Linear regression also showed that the probability of re-collapse of augmented vertebrae decreased by 14.4% for each quadrant increase in cement distribution. This provides strong evidence to support the repeated needle insertion technique^20^ in order to prevent re-collapse; the rationale of this technique is that repeated puncture into the unfilled area can distribute bone cement more uniformly and adequately. For new fractures, none of the evaluation methods in the present study showed a high predictive effect. The AUC of the 12-score method was the largest, but it was only 0.591, and linear regression showed a regression coefficient of only 0.033. These results suggest that bone cement distribution influences risk of new fracture much less than risk of re-collapse.

According to the literature, new fracture and re-collapse are related to a number of factors, including low BMD and BMI, presence of a cleft sign, reduction degree, vertebral augmentation, old fracture, thoracolumbar location, advanced age, and less bone cement^17–19,21,28–33^. However, which of these factors play stronger roles is unclear. Our study found patients with or without new fracture to differ in terms of age, number of augmented vertebrae, fracture cause, cleft sign and thoracolumbar location, while patients with or without re-collapse differed in number of augmented vertebrae, puncture method and thoracolumbar location. In this way, our analysis on which it is based may help clarify differential risk factors—besides bone cement distribution—for the two outcomes of re-collapse and new fracture.

There are several limitations of this study to consider. First, we applied methods based on calculation of scores rather than precise quantitative metrics in order to ensure ease of implementation. Second, since most of our patients experienced different degrees of pain during follow-up and therefore underwent X-ray examination, the proportions of patients with new fracture or re-collapse in our study may be relatively high. Our findings should be confirmed and extended in prospective studies. Third, we cannot rule out that quadrants in different positions of the vertebral body may differently influence risk of re-collapse and new fracture, although such differential influence should be minimal when bone cement has covered most of the quadrants of the vertebral body. Future studies should examine the differential influence of individual quadrants, depending on their location.

## Conclusion

Evaluating bone cement distribution based on X-ray images can be convenient and practical, and it can reliably predict new fracture and re-collapse of augmented vertebrae. The 12-score evaluation method showed good inter-rater consistency and strong ability to predict new fractures and re-collapse. A score of 10 with this method appears to be associated with the lowest risk of re-collapse and new fracture.
